# Inhibition of Advanced Glycation and Absence of Galectin-3 Prevent Blood-Retinal Barrier Dysfunction during Short-Term Diabetes

**DOI:** 10.1155/2007/51837

**Published:** 2007-04-12

**Authors:** Paul Canning, Josephine V. Glenn, Daniel K. Hsu, Fu-Tong Liu, Tom A. Gardiner, Alan W. Stitt

**Affiliations:** ^1^Centre for Vision Science, Queen's University Belfast, Royal Victoria Hospital, Grosvenor Road, Belfast BT12 6BA, Northern Ireland, UK; ^2^Department of Dermatology, School of Medicine, University of California, Sacramento, CA 95616, USA

## Abstract

Breakdown of the inner blood-retinal barrier (iBRB) occurs early in diabetes and is central to the development of sight-threatening diabetic macular edema (DME) as retinopathy progresses. In the current study, we examined how advanced glycation end products (AGEs) forming early in diabetes could modulate vasopermeability factor expression in the diabetic retina and alter inter-endothelial cell tight junction (TJ) integrity leading to iBRB dysfunction. We also investigated the potential for an AGE inhibitor to prevent this acute pathology and examined a role of the AGE-binding protein galectin-3 (Gal-3) in AGE-mediated cell retinal pathophysiology. Diabetes was induced in C57/BL6 wild-type (WT) mice and in Gal-3^−/−^ transgenic mice. Blood glucose was monitored and AGE levels were quantified by ELISA and immunohistochemistry. The diabetic groups were subdivided, and one group was treated with the AGE-inhibitor pyridoxamine (PM) while separate groups of WT and Gal-3^−/−^ mice were maintained as nondiabetic controls. iBRB integrity was assessed by Evans blue assay alongside visualisation of TJ protein complexes via occludin-1 immunolocalization in retinal flat mounts. Retinal expression levels of the vasopermeability factor VEGF were quantified using real-time RT-PCR and ELISA. WT diabetic mice showed significant AGE -immunoreactivity in the retinal microvasculature and also showed significant iBRB breakdown (*P* < .005). These diabetics had higher VEGF mRNA and protein expression in comparison to controls (*P* < .01). PM-treated diabetics had normal iBRB function and significantly reduced diabetes-mediated VEGF expression. Diabetic retinal vessels showed disrupted TJ integrity when compared to controls, while PM-treated diabetics demonstrated near-normal configuration. Gal-3^−/−^ mice showed significantly less diabetes-mediated iBRB dysfunction, junctional disruption, and VEGF expression changes than their WT counterparts. The data suggests an AGE-mediated disruption of iBRB via upregulation of VEGF in the diabetic retina, possibly modulating disruption of TJ integrity, even after acute diabetes. Prevention of AGE formation or genetic deletion of Gal-3 can effectively prevent these acute diabetic retinopathy changes.

## 1. INTRODUCTION

Breakdown of the inner blood-retinal barrier (iBRB) is a serious pathophysiological lesion in diabetic patients and if left untreated can lead to sight-threatening diabetic macular edema (DME), see [1, 2]. There are various approaches to quantification of iBRB dysfunction in patients, but irrespective of the technique employed [3, 4], it is established that this lesion occurs early in clinical diabetic retinopathy [5] and is associated with progression of the disease [6]. Breakdown of the iBRB is also a feature of experimental diabetes in animal models, being observed as early as 1-2-week postdiabetes induction in rodents [7, 8].

The precise mechanism of iBRB compromise during diabetic retinopathy remains incompletely elucidated but there are firm links with diabetes-mediated upregulation of the potent vasopermeability factor VEGF from the neural retina [9]. VEGF modulates loss of tight junction integrity or enhanced transport mechanisms in endothelial cells in the early stages of diabetic retinopathy [10, 11]. Upregulation of this growth factor occurs early in diabetes [12] which suggests that expression may be linked to acute hyperglycemia, alteration in retinal blood flow, and/or enhanced proinflammatory processes influencing retinal capillary function. Treatment of diabetic rodents with a range of agents that either modulate protein kinase C activation [13], prevent formation of reactive oxygen species (ROS) [14], or regulate aldose reductase activity [15, 16] can prevent diabetes-mediated rises in VEGF expression and prevent iBRB dysfunction.

The formation of advanced glycation end products (AGEs) is an important pathogenic mechanism in diabetic retinopathy. These adducts form on the amino groups of proteins, lipids, and DNA through nonenzymatic glycation reactions with glucose and also through highly reactive *α*-oxoaldehydes such as glyoxal, (GO), methylglyoxal (MGO), and 3-deoxyglucosone (3-DG) which can form AGEs very rapidly [17]. In support of clinical correlates, AGE-adducts are known to accumulate intracellularly in neurones and vascular cells, and extracellularly on basement membranes of diabetic retina [18–20] where they mediate pathophysiological changes [21–23]. Advanced glycation reactions also have relevant pathogenic effects on retinal vascular cells in vitro; responses that may, in part, be mediated through a range of AGE-binding proteins and receptors with defined proinflammatory signalling capacity and ability to modulate AGE-mediated pathophysiology [24–26].

In terms of iBRB function, AGEs are known to induce expression of the potent vasopermability agent VEGF in the retina in vivo [21, 27], or in retinal cells in vitro [28, 29]. Furthermore, these adducts can disrupt endothelial junctional complexes [30] and induce hyperpermeability in retinal capillary endothelial monolayers [31]. In vivo, AGEs have been linked to breakdown of the iBRB during diabetic retinopathy through infusion of preformed, highly modified “model AGEs” into nondiabetic animals which cause leakage of albumin and concomitant increases in VEGF mRNA expression [21, 32]. To date there have been no studies on the possibility of preventing acute VEGF upregulation or iBRB dysfunction using AGE inhibitors. Therefore, in the current study we have examined how AGEs, formed early in diabetes, could modulate vasopermeability factor expression in the diabetic retina and alter interendothelial cell tight junction integrity leading to iBRB dysfunction. Previous studies have shown a role for galectin-3 (Gal-3) in modulation of some AGE-mediated responses [26] therefore we have also investigated the contribution of this multifunctional lectin in AGE-mediated cell retinal pathophysiology in short-term diabetes.

## 2. MATERIALS AND METHODS

### 2.1. Animals and induction of diabetes

Both Gal-3^−/−^ and corresponding wild-type (WT) C57/BL6 controls at 6-8-week old were used in this study. All animals were housed and cared for in accordance with the ARVO Statement for the Use of Animals in Ophthalmic and Vision Research and to British Home office regulations. Gal-3 knockout mice (Gal-3^−/−^) were generated and backcrossed to C57/BL6 for 9 generations as previously described by Hsu et al. [33]. Male Gal-3^−/−^ and WT control animals were rendered diabetic with a single intraperitoneal injection of streptozotocin, 165 mg/kg in sterile filtered citrate buffer pH 4.6 (Sigma Chemical Company, Poole, England) as has been previously reported [34]. Nondiabetic WT animals were injected with an equivalent volume of citrate buffer alone. Blood glucose levels were measured one week postdiabetes induction and animals with glucose readings greater than 15 mmol/L were included in the study. A subset of diabetic animals subsequently received the AGE-inhibitor pyridoxamine (PM; pyridoxamine dihydrochloride, Biostratum Inc., Durham, NC, USA) administered orally in the drinking water (1 g/L). Water intake from all mice groups was monitored. Three weeks postdiabetes induction, and immediately prior to experimentation, animals were reweighted and blood glucose levels were remeasured to confirm diabetic status.

### 2.2. Assessment of AGEs in retina

Three weeks postdiabetes induction, 6 animals per group were euthanized by asphyxiation with carbon dioxide, the eyes enucleated, and retinas dissected under an operating microscope before being flash frozen in liquid nitrogen and stored at −80°C. The brain and the kidneys were also removed, flash frozen, and stored at −80°C. Protein was extracted by homogenising the retinal, brain, and kidney tissue extracts, respectively, in radioimmunoprecipitation buffer (RIPA; consisting of 0.5 M Tris-HCl, pH 6.8 containing 10% (w/v) SDS), over ice. Total protein was quantified by BCA assay (Pierce, MSC, Dublin, Ireland), and equivalent amounts loaded for AGE quantification by competitive ELISA using a polyclonal antibody to carboxymethyllysine (CML) (kind gift from Dr. Suzanne Thorpe, University of South Carolina, Columbia, SC, USA), see [22].

### 2.3. Assessment of blood-retinal barrier breakdown

Plasma albumin leakage from the diabetic retinal vasculature and hence iBRB dysfunction were quantified in using Evans blue in accordance with the published protocol [8] with modifications as outlined by Brankin et al. [35]. Briefly, Evans blue dye (Sigma), was dissolved in PBS (30 mg/mL), sonicated for 5 minutes, and filtered through a 0.45 *μ*m filter. Nondiabetic, diabetic, and PM-treated diabetic wild-type and Gal-3^−/−^ mice (*n* = 8/group) were anaesthetized with isofluorane and Evans blue dye intravenously administered by tail vein injection (26 g, Venisystems Ltd., Abbot Ireland Ltd., Sligo, Eire) at a dose of 45 mg/kg in a volume of 200 *μ*L upon which mice were seen to turn blue, and this was used as a confirmation that the dye had been taken into the bloodstream.

Quantification of dye leakage into the neuropile was assayed as previously described [8]. Briefly, 3 hours after Evan's blue injection the mice were perfused using citrate buffer at a pressure of 120 mm/Hg (for up to 2 minutes). Both eyes were enucleated and retinas were removed. After determination of the wet weight, the retinas were completely dried by placing in a Speed-Vac overnight at 60°C. Retinal dry weights were subsequently determined, and retinas then crushed in 120 *μ*L formamide at 70°C for 18 hours, in order to remove Evan's blue. After this time, the extract was centrifuged with a filter centrifuge tube at 15 000 rpm for 30 minutes in order to remove retinal debris. The filtrate was subsequently read on the spectrophotometer at an absorbance of 620 nm, the absorbance maximum for Evan's blue, and 740 nm, the absorbance minimum. The concentration of dye in the extracts was calculated from a standard curve of Evan's blue in formamide. The BRB breakdown was calculated as outlined by Qaum et al. [8].

For visual assessment of leakage, animals were deeply anaesthetized using ketamine/xylazine and 40 kd FITC-Dextran (Sigma) (30 mg/mL in sterile PBS) injected into the left ventricle. The tracer was allowed to circulate for ∼2 minutes and the eyes were then enucleated and immediately fixed in 4% PFA. The eyes were fixed overnight in 2% paraformaldehyde and the following day the retinas were dissected off, cut in a Maltese cross-configuration, and flat-mounted onto glass slides. The retinas were then viewed using epifluorescence.

### 2.4. Quantification of retinal VEGF mRNA and protein

Animals were treated as described previously and eyes enucleated immediately following sacrifice (*n* = 6/group). Retinas were dissected away from the posterior eye cup and placed in an RNA stabilisation reagent (RNAlater, Ambion, Austin, Tex, USA) and stored at 4°C. Total retinal RNA was extracted with Tri-reagent (Sigma) by standard isopropanol: chloroform precipitation as described in the manufacturer's instructions. The resulting RNA pellets were washed twice with 75% ethanol, and resuspended in 30 *μ*L diethyl pyrocarbonate (DEPC) treated water. RNA integrity and quality were confirmed by analysis of 260 : 280 nm absorbance ratio, and visualisation of ribosomal 28 s and 18 s bands on a 1% agarose gel. cDNA was synthesized from 2 *μ*g retinal RNA using a reverse transcriptase cDNA synthesis kit (ImProm II, Promega, MSC, Dublin, Ireland) according to manufacturer's instructions on an automated Applied Biosystems 2720 Thermal Cycler.

cDNA was diluted twenty-fold prior to PCR amplification. Real-time PCR analysis was performed for quantitative analysis of mRNA expression. A 189 bp-fragment of VEGF cDNA was amplified with murine-specific primers (forward: 5′ TTACTGCTGTACCTCCACC 3′; reverse: 5′ ACAGGACGGCTTGAAGATG 3′). In addition, primers to amplify a 100 bp-fragment of the housekeeping gene 28 s ribosomal RNA were used: (forward 5′ TTGAAAATCCGGGGGAGAG 3′; reverse 5′ ACATTGTTCCAACATGCCAG 3′). Real-time RT-PCR was performed with an ABI Prism 7000 Sequence Detection system. PCR was performed in a 96-well plate format (ABgen, Rochester, NY, USA), in a 20 *μ*L volume, containing 0.5 *μ*M primers, reaction buffer, 2.5 mM MgCl_2_, dNTPs, *Taq* DNA polymerase (hotstart), and SYBER green I fluorescent dye (Qiagen, Crawley, UK). Amplification involved an initial 15-minute denaturation step, followed by up to 45 cycles of a 95°C denaturation for 15 seconds, 52–58°C annealing for 20 seconds, and 72°C for an appropriate extension time (5–25 seconds). Fluorescence of the green dye that was bound to the PCR product was detected at the end of each extension period and the specificity of the amplification reactions confirmed by melting curve analysis and subsequent agarose gel electrophoresis. PCR amplification reactions were performed in triplicate on material from at least two independent reverse transcription reactions. Quantification data was analysed by the delta Ct method [36], and normalised to the housekeeping gene 28 s ribosomal RNA.

For VEGF protein quantification, retinas were freshly dissected, and placed in 200 *μ*L RIPA buffer supplemented with protease inhibitor cocktail (Roche, Mannheim, Germany). Retina was homogenised with a plastic pestle and hand held battery rotor, and sonicated. The lysate was centrifuged at 13 000 rpm for 5 minutes, and the resulting supernatants assayed for VEGF activity using the Quantikine Mouse VEGF Immunoassay kit (R&D Systems) as per the manufacturer's instructions.

### 2.5. Immunohistochemistry

The retinal vasculature was visualised by reaction with biotinylated isolectin B4 from *Griffonia Simplicifolia* (Sigma-Aldrich Ltd., Gillingham, UK) at 50 ng/mL followed by streptavidin-Alexa 568 (Molecular Probes Europe BV, Leiden, Netherlands). Retinal flatmounts were washed extensively and mounted in Vectasheild (Vector Laboratories Ltd., Peterborough, UK). Images were acquired using an Olympus BX60 fluorescence microscope (Olympus UK Ltd., London, UK) fitted with a MicroRadiance confocal scanning laser microscope (CSLM) (Bio-Rad Laboratories, Hercules, Calif, USA).

For methylglyoxal (MG)-derived AGE immunolocalization, one eye from 5 animals from each treatment regime was fixed in 4% PFA for 4 hours and then washed extensively over a 4-hour period after which the anterior segment-lens complexes were removed and the posterior eye cups relaxed by placing 4 radial cuts from the retinal periphery to points within 1 mm from the optic disc. The posterior eye cups were permeabilised and nonspecific immunoreactive sites blocked for 16 hourrs at 4°C in PBS containing 0.5% Triton X-100 (TX-100), 5% normal goat serum and mouse-on-mouse reagent (Vector Laboratories, Youngstown, Ohio, USA). Monoclonal anti-MG modified protein antibody (kindly donated by Dr. Ryoji Nagai, Kumamoto University, Kumamoto, Japan) or control mouse IgG (Sigma) was added to the retinas overnight at 4°C at 1 : 1000 dilution in PBS containing 0.5% TX-100. The retinas were then blocked in 5% NGS in permeabilising buffer, washed extensively, and exposed to antimouse-488 nm Alexa (Molecular Probes Inc., Eugene, Ore, USA), diluted 1 : 500 in PBS containing TX-100 for 3 hours at 4°C. The retinas were then washed extensively, mounted in Citifluor (Agar Scientific Ltd., Essex, England) on microscope slides and immunofluorescence detected by CSLM. Gain settings were kept constant between specimens during digital confocal image capture, in order to compare the intensity of immunoreactivity in the retina between treatment groups.

For occludin immunolocalization, 5 eyes from animals from each treatment group were enucleated and fixed in 70% ethanol for 30 minutes at 4°C. Following this initial fixation, eyes were transferred to acetone that had been prechilled at −20°C, and fixed for further 3 minutes. Retinas were dissected, blocked, and permeabilised with blocking reagent (PBS, 0.5% Triton X-100, and 5% goat serum) overnight at 4°C, prior to incubation with mouse antioccludin primary antibody (Zymed Laboratories, South San Francisco, Calif, USA) (1 : 3000 dilution in blocking reagent) for two days at 4°C. Retinas were washed six times in PBS and incubated with a goat antimouse Alexa 488 nm secondary antibody (1 : 2000 dilution in block solution) (Invitrogen, Paisley, UK) for 2 hours at room temperature. Retinas were washed for further six times in PBS (20 minutes each, as previously) before flat mounting on microscope slides by making four radial cuts in a Maltese cross-configuration. Flat-mounted retinas were visualised using CSLM as outlined above.

## 3. RESULTS

### 3.1. Diabetes and formation of AGEs

Blood glucose levels were elevated in the diabetic groups when compared to nondiabetic (*P* < .001) and there was no difference between WT and Gal-3^−/−^ groups (Table 1). PM had no significant influence on hyperglycaemia in either Gal-3^−/−^ or WT mice (Table 1). Diabetic WT and Gal-3^−/−^ also exhibited characteristic loss of weight when compared to their respective nondiabetic counterparts (Table 1).

As determined by competitive ELISA, CML levels were modestly but significantly elevated in the retinas of WT diabetic mice when compared to nondiabetic controls (*P* < .05). Nondiabetic Gal-3^−/−^ animals had significantly less retinal CML when compared to WT control (*P* < .05). Diabetes increased this CML-immunoreactivity in Gal-3^−/−^ animals. In all cases, PM had no appreciable influence on this parameter (Table 1). For reference, kidney and brain from animals recruited to this study were also assessed for AGEs. It was determined that CML was elevated in diabetes in WT and Gal-3^−/−^ mice. In kidney, absence of Gal-3 reduced the CML content after 3-week diabetes (*P* < .05).

**Figure 1:**
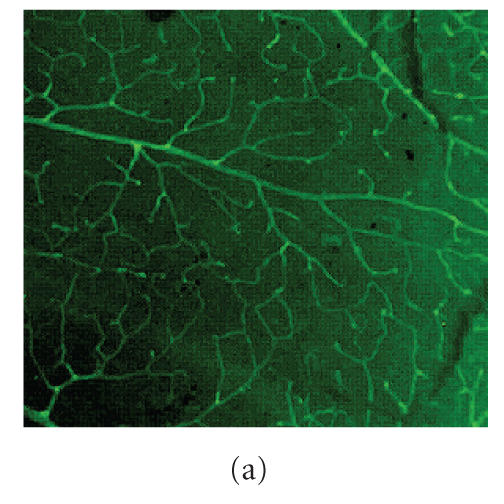

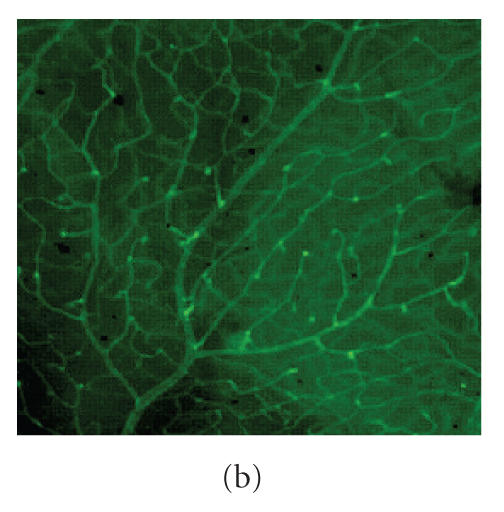

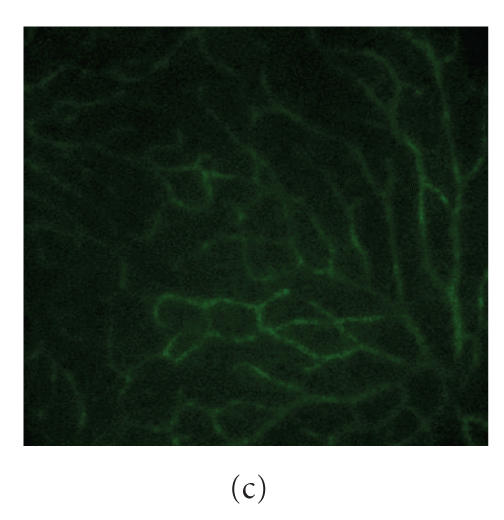

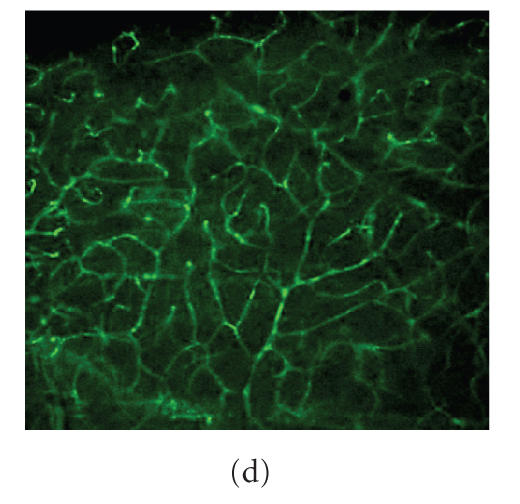

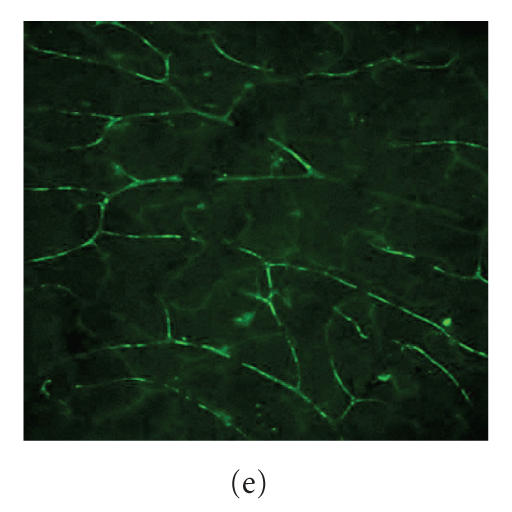

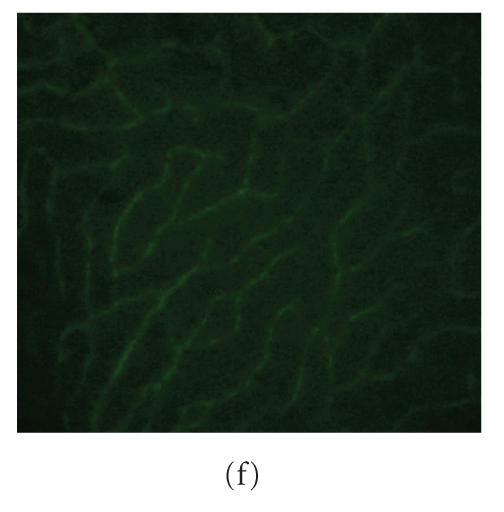
AGE accumulation in the diabetic retina. Lectin staining shows the vascular tree in (a) nondiabetic and (b) diabetic animals. At such an acute time frame of diabetes, there is no appreciable difference in density of capillaries. Also on retinal flat mounts, an antibody that recognizes MG-derived AGEs shows modest immunoreactivity in the retinal-microvasculature in nondiabetic mice (c). Diabetic mice (d) show considerably more intense AGE labelling, again largely within the retinal-blood vessels which are only partially reduced in diabetics treated with PM (e). Controls show only background immunoreactivity (f). Original magnification x200.

Isolectin staining showed the microvascular tree in the retinal flatmounts (Figures 1(a) and 1(b)). Immunohistochemistry using a monoclonal antibody that recognises MG-derived adducts showed localization to the intraretinal microvasculature, both in terms of the superficial and deep capillary plexi. In all diabetic mice examined there was marked increase in vascular staining when compared to nondiabetic controls (compare Figure 1(c) with Figure 1(d)). PM-treatment reduced this MG-adduct immunofluorescence (Figure 1(e)) and in all controls (in which mouse-on-mouse block was used) negative staining was observed (Figure 1(f)).

### 3.2. Blood-retinal barrier function

**Figure 2:**
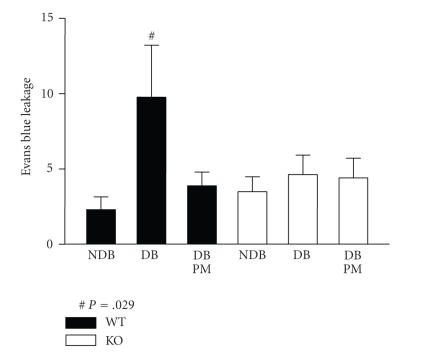
Inner blood-retinal barrier (iBRB) function in diabetic WT and Gal-3^−/−^ mice. As determined by Evan's blue assay, diabetic animals (DB) show a significant increase in BRB dysfunction when compared to nondiabetic (NDB) controls. Pyridoxamine (PM) treated diabetic animals do not show this vasopermeability response and are more comparable to nondiabetic controls. Gal-3^−/−^ fails to demonstrate diabetes-induced barrier dysfunction.

Breakdown of the BRB as determined by Evans blue dye leakage, was up to 4-fold greater after 2-week diabetes in WT mice compared to nondiabetic controls (*P* < .005). PM-treatment of diabetic animals significantly prevented this vasopermeability response, to the extent that there was no significant difference between nondiabetic and PM-treated diabetic WT mice (Figure 2). Gal-3^−/−^ mice failed to demonstrate any diabetes-mediated increase in BRB dysfunction which contrasted markedly with their WT counterparts (Figure 2).

### 3.3. Tight junction integrity

**Figure 3:**
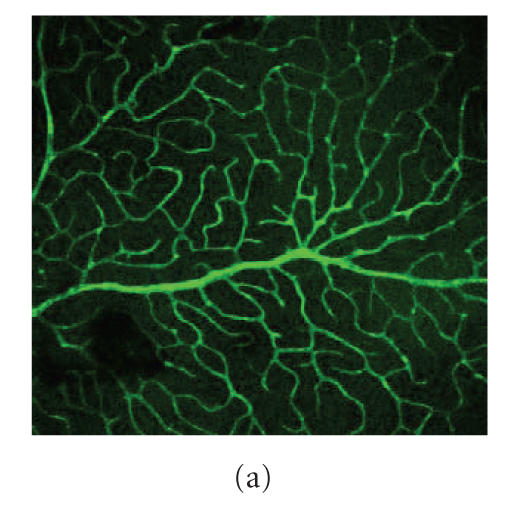

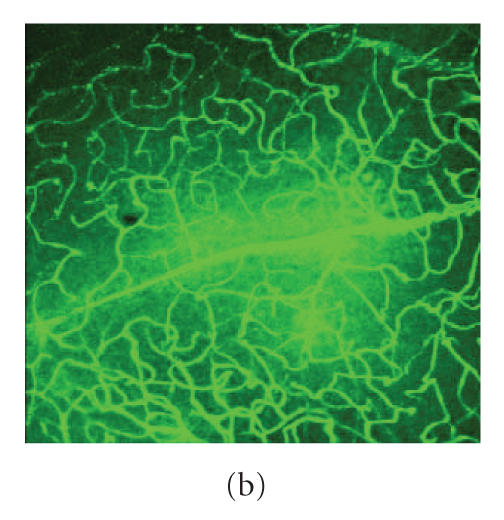

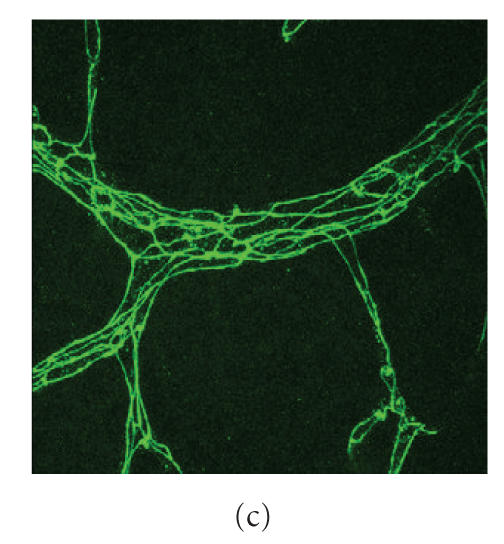

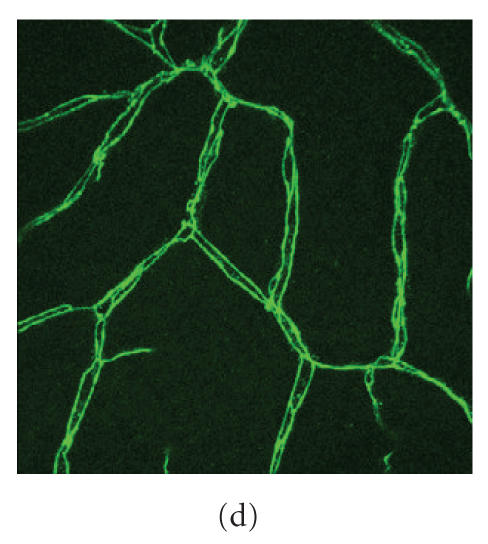

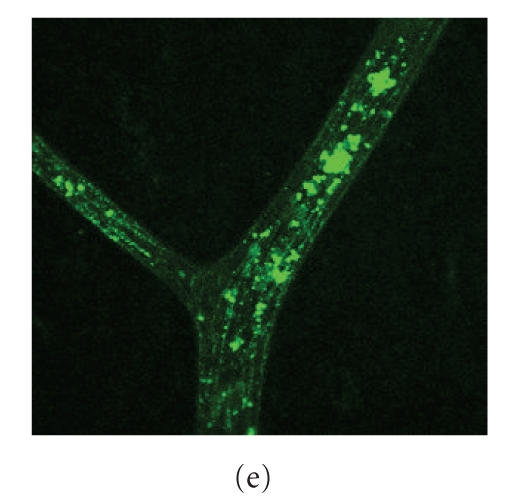

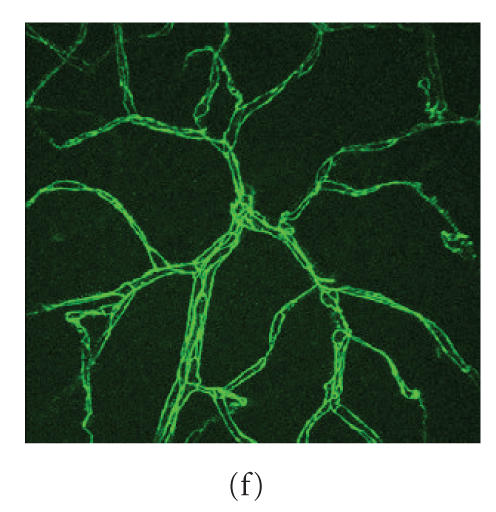

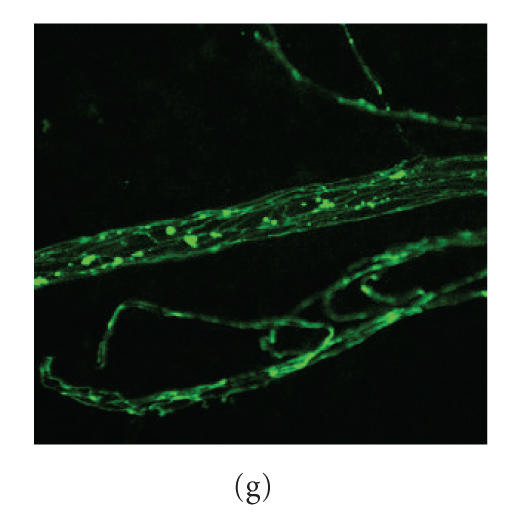

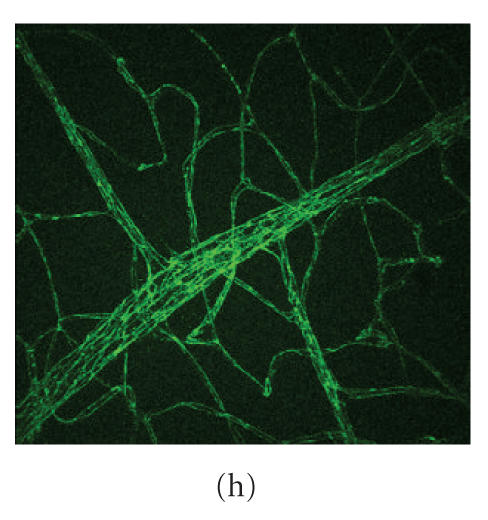
Diabetes alters tight junction integrity in retinal capillaries. Fluorescein dextran angiography on retinal flat mounts shows integrity of the retinal microvasculature in nondiabetic mice (a) while there is a clear leakage of tracer (∼40 kd) into the retinal neuropile in 2-week diabetic animals (b) (original magnification x100). Fluorescent immunostaining for occludin-1 of retinal flat mounts from nondiabetic WT mice demonstrates plasma membrane localization at the tight junctions (TJs) of the endothelium (c). By contrast with diabetic animals, there is aggregation of occludin-1 in the cytoplasm of endothelial cells rather than at the plasma membrane (e). PM treatment of diabetic mice only partially prevents disruption of the junctional integrity (g). Nondiabetic (d), diabetic (f), and PM-treated Gal-3^−/−^ mice (h) show none of the diabetes-induced changes observed in WT counterparts (original magnification x200).

Leakage of FITC-dextran of 40 kd was evident in diabetic retinas while nondiabetic controls showed no leakage of this tracer (compare Figures 3(a) and 3(b)). Diabetes also had a profound effect on integrity of tight junctions, as determined by immunolocalization of the junctional complex component protein occludin in retinal flatmounts. Immunostaining for occludin-1 demonstrated integrity of the tight junctions between arterial, capillary, and venous endothelium in the nondiabetic WT animals (Figure 3(c)). Diabetes significantly altered this configuration, with less defined occludin-immunoreactivity in TJ complexes, showing a cytoplasmic staining pattern rather than being localised at the plasma membrane (Figure 3(e)). PM treatment seemed to restore some of this integrity in diabetic animals (Figure 3(g)). In contrast to WT counterparts, the retinal microvasculature of Gal-3^−/−^ demonstrated integrity of tight junctions, not only in nondiabetic controls but also in diabetic and nondiabetic animals treated with PM (Figures 3(d), 3(f), and 3(h)).

### 3.4. VEGF expression

**Figure 4:**
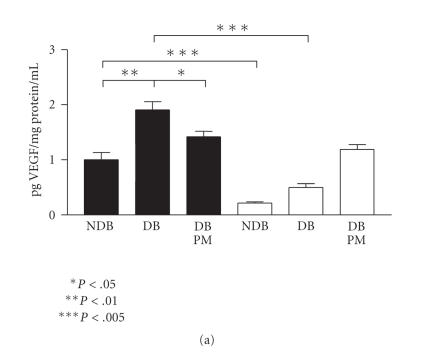

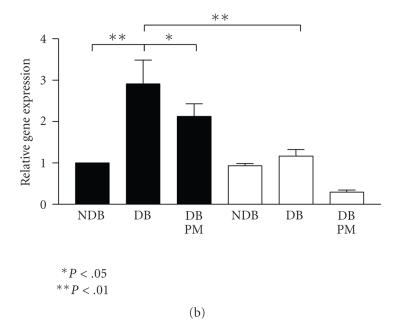
VEGF protein and mRNA expression in diabetic Gal-3^−/−^ and WT mouse retina. In WT mice (black bars) VEGF expression is significantly increased in diabetic (DB) retina—in terms of both protein (a) and mRNA expression (b) when compared to nondiabetic controls (NDB). PM-treatment significantly reduces VEGF expression in diabetic mice. In Gal-3^−/−^ mice (white bars), retinal expression levels are not as dramatically increased by the presence of diabetes as evidenced in WT mice.

ELISA showed that after only two weeks of diabetes in WT mice VEGF peptide was increased twofold over nondiabetic controls (*P* < .01) (Figure 4(a)). PM-treatment showed a significant, albeit incomplete, reduction in this diabetes-mediated increase (*P* < .05). The Gal-3^−/−^ animals had a much lower baseline level of VEGF when nondiabetic WT was compared with the transgenic counterpart (*P* < .001). VEGF levels were increased upon induction of diabetes in the Gal-3^−/−^ mice and while the magnitude of change was comparable to WT, the VEGF levels in Gal-3^−/−^ were still lower than nondiabetic WT. PM treatment of Gal-3^−/−^ diabetic mice increased VEGF relative to diabetic counterparts (*P* < .01) (Figure 4(a)).

For all relative mRNA expression data, data was normalised to the housekeeping gene ribosomal 28 s, and results were expressed relative to the levels in the WT nondiabetic control group. VEGF mRNA fluctuation correlated closely with the trend seen for Evans blue leakage between the various treatment groups of animals. VEGF mRNA expression was almost three-fold higher in the untreated WT diabetic control group compared to the nondiabetic controls (*P* < .01) (Figure 4(b)). PM treatment reduced this increase in diabetic VEGF mRNA levels (*P* < .05). In the corresponding Gal-3^−/−^ animals, the diabetes-induced increase in retinal VEGF mRNA expression was less pronounced than in WT. Nevertheless, PM treatment decreased the VEGF mRNA expression levels to less than those measured in the nondiabetic control animal group (*P* < .01) (Figure 4(b)).

## 4. DISCUSSION

Breakdown of the iBRB is an established lesion in clinical diabetes. Abnormal microvascular leakage can occur early after establishment of diabetes although it remains uncertain how this relates to more severe, sight-threatening macular oedema. For study of iBRB dysfunction in experimental diabetes most focus has been placed on short-term retinopathy models in rodents where acute phase vasopermeability is evident after only 2-3-week diabetes [7, 8]. Various studies have shown that manipulation of adhesion molecules, proinflammatory cytokines, and nitric oxide can prevent this lesion within an acute time frame [7, 8, 37, 38]. The current study has demonstrated that AGE inhibition by PM, soon after diabetes induction, also prevents iBRB dysfunction with concomitant VEGF upregulation and loss of tight junction integrity.

Advanced glycation is an important pathogenic factor in diabetic retinopathy and most other vascular complications of diabetes [39]. AGE-modifications are usually considered to take months or years to accumulate in diabetic tissues, but it is now clear that these adducts can also form rapidly during hyperglycaemia [19]. Exposure of retinal glial and vascular cells to high glucose in vitro can induce significant AGE formation after 7–10 days [40, 41], leading to modifications that have profound pathogenic consequences [41–43]. Hyperglycaemia-linked formation of highly reactive *α*-oxaloaldehydes is a major source of these rapidly formed intra- and extracellular adducts such as N*ɛ*-(carboxymethyl)lysine (CML), N*ɛ*-(carboxyethyl)lysine (CEL), and MG-derived hydroimidazolone [17]. Indeed, MG is raised in serum from diabetic patients [44] and the immunolocalization data in the current study suggests that AGEs derived from this dicarbonyl also accumulate in the retinal microvasculature of acutely diabetic mice. MG-derived AGEs have been shown to occur in retinal microvascular endothelial cells exposed for 10 days to high glucose in vitro which is within the time boundaries of the current in vivo study [40]. MG-modifications have been shown to induce profound cell responses such as altering transcriptional regulation of vasoactive growth factors [41]. MG-modifications of the basement membrane also induce dysfunctional responses in retinal microvascular endothelial cells [45] and it is reasonable to assume that rapid formation of both intracellular and extracellular MG-modifications in vivo has pathogenic implications for endothelial dysfunction and iBRB compromise during acute diabetes.

Inhibitors of advanced glycation have shown efficacy in reducing retinal microvascular lesions in diabetic animal models [22, 23, 46]. With direct relevance to the current study, PM has been shown to have beneficial effects by preventing CML formation and subsequent lesion formation in diabetic retinopathy in a rat model over a 7-month time frame [22] with an efficacy that is comparable to other diabetic complications [47]. We have now demonstrated that PM is also effective against iBRB dysfunction within an acute treatment regime. The mechanism of this action is probably linked to the ability of PM to prevent MG-derived AGE formation as evidenced by the present immunohistochemistry data in combination with previous studies that have demonstrated PM efficacy against MG-derived AGEs [48]. Perhaps unsurprisingly there was no effect of PM treatment on non-MG-derived CML formation over this short time frame in retina, kidney, or brain which contrasted with previously reported long-term outcomes [22]. It is interesting that PM could have short-term benefit over a significant diabetic retinopathy lesion and, while more investigation is required, this could indicate applicability for anti-AGE strategies over a shorter time frame than previously thought.

AGEs have been linked to increases in retinal VEGF expression although the precise mechanism for this remains uncertain [49]. Since the major sources of VEGF expression in the retina are Muller glia and ganglion cells it would seem that these cells are influenced by AGE adducts either through endogenous formation or as a response to exposure from leaked AGE-modified serum proteins. We have previously shown that Muller glia and ganglion cells accumulate AGE adducts (CML) over 7-month diabetes [22], but following more acute exposure to hyperglycaemia it is possible that these cells could form significant AGEs and respond by upregulating VEGF. MG-modification of the transcriptional corepressor protein mSin3A has been shown as the mechanism whereby retinal levels of the growth factor angiopoietin-2 are upregulated in Muller glia in vitro [41]. It is interesting that most of the MG-derived protein modifications in the inner retina were vascular-localised with lesser immunoreactivity in the Muller glia. The reason for this is unclear but it is possible that acute hyperglycaemia has most immediate impact on the endothelium and that longer periods of high glucose exposure are required to raise intracellular levels of AGEs in the retinal neuropile. The chemical nature, time frame of formation, and cellular localization of various AGEs are important parameters to establish and such studies are currently ongoing in our laboratory.

The current investigation indicates that iBRB dysfunction in diabetes is modulated by the presence of Gal-3. This multifunctional protein has a diverse range properties linked to its carbohydrate binding capacity and may be involved in immune processes and neoplastic disease [50]. Gal-3 also has AGE-binding properties and several diabetes-related pathophysiological responses are mediated, at least in part, by Gal-3 (also referred to as AGE-R3 [51]). The role of Gal-3 as an AGE-binding protein with links to diabetic microvasculopathy has been previously demonstrated [52–54] and this protein also plays a significant role in AGE-related pathophysiology during diabetic retinopathy. We have previously demonstrated that Gal-3^−/−^ mice or neutralisation with a Gal-3 antibody reversed the inhibition of retinal angiogenesis by diabetic sera or exogenous AGEs [26]. The presence of Gal-3 appears to be required for iBRB dysfunction during acute diabetes where it may modulate cell responses to AGEs. How this occurs is uncertain, especially when one considers that exogenous AGEs (whether occurring as modified serum proteins or as substrate-immobilized cross-links) are not significantly different in 2-week diabetics and nondiabetic controls. It remains possible that Gal-3 could alter vascular cell function independent of AGE binding or that the protein could bind to intracellular adducts illicit cell stability against AGE-induced changes.

It is entirely possible that Gal-3 could modulate barrier dysfunction during diabetes by mechanisms that are additional to, or distinct from, AGE binding. Beyond an AGE binding role, various possibilities can be suggested for Gal-3 modulation of iBRB dysfunction in diabetes. Gal-3 binds many substrate proteins including collagen IV, fibronectin, and elastin and is a main element in promoting cell adhesion [50]. It is also interesting to note that this protein has a distinct role in immune responses and it has been shown to promote mast cell activation and proinflammatory signals in a number of tissues, characterised by promoting adhesion of neutrophils and monocytes to endothelial cells [50, 55]. Indeed, Gal-3^−/−^ mice show reduced inflammatory responses during peritonitis [56] while macrophages from these animals have attenuated phagocytic capacity when compared to wild-type controls [57]. iBRB compromise in diabetic retinopathy is not necessarily separate from these proinflammatory processes, indeed, during diabetes the retinal microvasculature shows enhanced expression of endothelial ICAM-1/VCAM-1 adhesion molecules and promotion of capillary leukostasis [37, 38]. AGEs have been shown to promote these responses in the murine retina [21, 32]. Investigations into the links between Gal-3, retinal capillary leukostasis, and iBRB dysfunction are ongoing. More research needs to be conducted on Gal-3 and its ability to regulate pathogenic responses in diabetic retinopathy, perhaps with respect to interaction with the well-characterised AGE receptor RAGE [54].

## Figures and Tables

**Table 1:** Metabolic parameters and AGE accumulation in tissues from diabetic (DB) and nondiabetic (NDB) mice. Blood glucose levels are significantly higher in the diabetic groups when compared to nondiabetic controls. There is no difference between WT and Gal-3^−/−^ mice, nor does pyridoxamine (PM) have any effect on hyperglycaemia. Diabetic WT and Gal-3^−/−^ mice also show characteristic loss of weight when compared to their respective nondiabetic counterparts. Retinal CML-immunoreactivity is elevated in WT diabetic mice when compared to nondiabetic controls (*P* < .05). Nondiabetic Gal-3^−/−^ animals have significantly less retinal CML when compared to WT control and PM has no appreciable influence on CML accumulation over this 2-week time frame. As reference tissues, kidney and brain CML-immunoreactivities are elevated in diabetes in WT and Gal-3^−/−^ mice (± standard deviation).

			CML (pg CML/mg protein)		
			
	Weight	Blood glucose	Brain	Kidney	Retina

WT NDB	27.5 ± 2.08	15.85 ± 5.44	58.73 ± 8.64	299.57 ± 86.0	81.77
WT DB	19.15 ± 2.87*	30.99 ± 5.97**	60.60 ± 6.26	341.28 ± 137.63	87.42*
WT DB PM	19.10 ± 4.51*	28.23 ± 8.26**	60.29 ± 8.54	348.29 ± 115.44	89.65*
Gal-3^−/−^ NDB	24.24 ± 1.97	12.46 ± 4.78	64.13 ± 3.98	386.19 ± 135.17	72.09*
Gal-3^−/−^ DB	22.14 ± 2.07	30.46 ± 4.05	69.66 ± 4.99	310.79 ± 224.41	87.18
Gal-3^−/−^ DB PM	20.50 ± 1.78	28.76 ± 6.11	66.45 ± 5.68	281.73 ± 48.90	89.14

*P < .05.

**P < .001.
